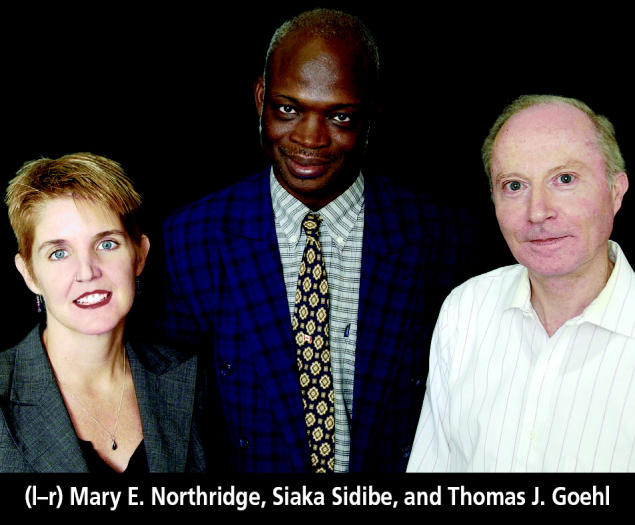# Editorial: Environment and Health: Capacity Building for the Future

**DOI:** 10.1289/ehp.112-1247638

**Published:** 2004-11

**Authors:** Mary E. Northridge, Siaka Sidibe, Thomas J. Goehl

**Affiliations:** Editor-In-Chief, *American Journal of Public Health*, New York, New York, E-mail: men11@columbia.edu; Editor-in-Chief, *Mali Médical*, Faculté de Médecine, de Pharmacie et d’Odontostomatologie, Bamako, Mali, E-mail: sidibes@voila.fr; Editor-in-Chief, *EHP*, Research Triangle Park, North Carolina, E-mail: goehl@niehl.nih.gov

In October 2002, the World Health Organization (WHO) sponsored a workshop in Geneva, Switzerland, to address the problems faced by medical journals in the developing world regarding their efforts to provide critical, timely health information to local health practitioners and research scientists. Of major concern is the unavailability of the results of medical research from developing nations, which is published in international journals, to those who are living and working in developing nations. If scientific knowledge is to be used to improve public health and the environment, then it must be accessible to the local health professionals who need it most. A specific outcome of this workshop was the creation of the Forum for African Medical Editors (FAME) with 12 inaugural African medical editors, both anglophone and francophone. While the WHO is a major sponsor of FAME, other participating organizations, institutions, journals, and associations offer various forms of assistance.

In September 2003, the U.S. National Institutes of Health (NIH)— specifically, the National Library of Medicine (NLM), the Fogarty International Center, and the National Institute of Environmental Health Sciences (NIEHS)—sponsored a meeting in London at the offices of the *British Medical Journal* (*BMJ*). The primary objective was to discuss the partnership of four sub-Saharan African medical journals with five Northern Hemisphere medical journals as a mechanism to enhance the quality and credibility of the African journals and thereby attract high-level research.

Identified steps needed to enhance the quality of the African journals included providing training for editors in improving sustainability and publishing regularity, improving the peer-review process by identifying experienced reviewers willing to serve and mentoring new reviewers, and offering local researchers guidance in preparing research papers for publication. Ideas for improving the credibility of the African journals included having respected scientists from multiple countries serve on the journals’ editorial boards, earning inclusion in major indexing databases such as the NLM’s MEDLINE, and exploring ways to share journal content, for example, by copublishing peer-reviewed research articles of high importance to the people in the area served by regional journals. This latter approach would have the added benefit for researchers and health practitioners in developed countries of making important regional research results more available in the international literature.

In May 2004, a contract was awarded to the Council of Science Editors to manage the funds for a pilot project intended to build the capacity of the four sub-Saharan African journals as per the thoughts generated at the London meeting. The African journals were selected because all of their editors are founding members of FAME and thus are committed to enhancing the capacities of their journals as well as other sub-Saharan medical journals. In addition, the African journals are published in countries that have active NIH-sponsored research and are also part of the communication network developed by the NLM for the Multilateral Initiative on Malaria. The Northern Hemisphere journals were selected on the basis of their missions and commitment to advancing public health and the environment in developing regions of the world. The following four journal partnerships have been established: *a*) *African Health Sciences* with *BMJ*; *b*) *Ghana Medical Journal* with *The Lancet*; *c*) *Malawi Medical Journal* with the *Journal of the American Medical Association*; and *d*) *Mali Médical* with *Environmental Health Perspectives* (*EHP*) and the *American Journal of Public Health*. The last partnership—ours—is the only one involving two Northern Hemisphere journals and the only one involving a francophone journal.

If scientific knowledge is to be used to improve public health and the environment, then it must be accessible to the local health professionals who need it most.

In September 2004, the three of us met in Research Triangle Park, North Carolina, to begin working toward the successful completion of our contract tasks, which were as follows:

1. To identify the equipment and facility needs of the *Mali Médical* and then provide computer hardware and software to the publishing offices, along with initial training for editorial office personnel
2. To identify the editorial needs of the *Mali Médical* through mutual site visits by the partnering editors-in-chief to observe editorial and publishing practices
3. To provide author/reviewer training via workshops, emphasizing international standards for writing and systematic approaches for reviewers, open to all FAME members at scheduled scientific/medical meetings in Africa
4. To provide training and support for a managing editor/business manager in establishing business plans for effective, sustainable publishing operations through technical consultation and through a workshop in Africa open to all FAME members
5. To develop and maintain a website that would permit online publication of the *Mali Médical*
6. To establish internships for representatives of the *Mali Médical* at the editorial offices of *EHP* and the *American Journal of Public Health*
7. To commission four systematic reviews on topics relevant to sub-Saharan Africa to be published in partnering African journals in both English and French.

Over the next several years, we plan to evaluate the success of our capacity-building initiative using the following indicators: progress toward indexing the *Mali Médical* in MEDLINE, numbers of articles submitted and published, numbers and effectiveness of local peer reviewers, and timeliness of publication. If our journals and sponsoring organizations are to fulfill our common missions of working to improve the public’s health and achieving equity in health status for all, then our nascent partnership is a viable means toward this end. Our collective hope is that all three journals will better realize their potential to serve as vehicles for progressive change through increased understanding, collaboration, insight, and connections between the environment and health in the developed and developing world. Look for regular updates from us published simultaneously in all three journals in both English and French. We aim to hold one another accountable in fulfilling our assigned tasks and enlisting other partners in our rewarding struggle to find creative and practical solutions that eliminate past and present health inequalities and protect the environment for future generations.

## Figures and Tables

**Figure:**